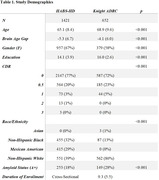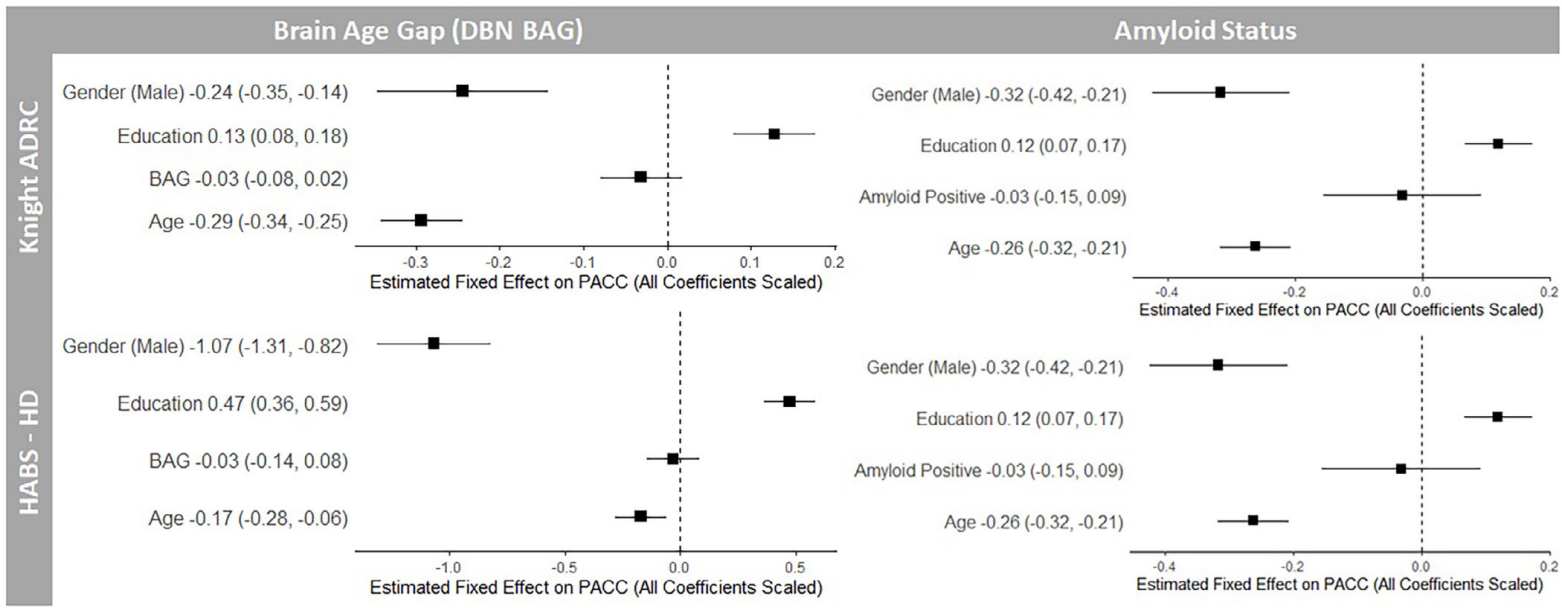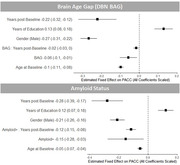# Brain Age Gap has a Small Association with Cognition in Cognitively Normal Older Adults

**DOI:** 10.1002/alz.091574

**Published:** 2025-01-09

**Authors:** Charlotte Best, Kalen Petersen, Peter R Millar, Nicole S. McKay, Andrew J. Aschenbrenner, Brian A. Gordon, David A. Balota, Suzanne E. Schindler, Tammie L.S. Benzinger, Meredith N Braskie, Arthur W. Toga, Sid E. O'Bryant, Beau Ances, Julie K. Wisch, the HABS‐HD Study Team

**Affiliations:** ^1^ Michigan State University, East Lansing, MI USA; ^2^ Washington University School of Medicine, St. Louis, MO USA; ^3^ Washington University in St. Louis School of Medicine, St. Louis, MO USA; ^4^ Washington University School of Medicine in St. Louis, St. Louis, MO USA; ^5^ Stevens Neuroimaging and Informatics Institute, Los Angeles, CA USA; ^6^ University of Southern California, Los Angeles, CA USA; ^7^ University of North Texas Health Science Center, Fort Worth, TX USA

## Abstract

**Background:**

Brain Age Gap (BAG) describes the difference between predicted brain age and chronological age, where a higher BAG suggests accelerated brain aging. It is estimated using machine learning techniques. The Preclinical Alzheimer Cognitive Composite (PACC) is a combination of neuropsychological tests employed in Alzheimer Disease (AD) research. It is sensitive to subtle change in cognition across the preclinical period of AD. BAG associates with change in Clinical Dementia Rating (CDR), but its relevance to subtle cognitive decline as compared to a biomarker of preclinical AD (amyloid PET) is unknown.

**Method:**

Longitudinal data were collected from the Knight AD Research Center (ADRC; N = 652, mean duration of cognitive follow‐up = 9.3 years) and cross‐sectional data from the Health & Aging Brain Study‐Health Disparities (HABS‐HD; N = 1421). All participants were CDR=0. We estimated BAG using DeepBrainNet, adjusting for age and scanner. Amyloid PET was collected using [18F]florbetaben in HABS‐HD and both [18F]florbetaben and [11C]PiB (converted to Centiloids) in the Knight ADRC. We calculated PACC using previously published methods, relying on different tests in each cohort. We evaluated cross‐sectional relationships between PACC and both BAG and amyloid PET in both cohorts, and longitudinal relationships in the Knight ADRC cohort. Analyses were adjusted for age, education, and gender.

**Result:**

There was no cross‐sectional association between PACC and either BAG or amyloid status. Longitudinally, there were baseline differences by both BAG (β_scaled_ = ‐0.06, p = 0.01) and amyloid status (β_scaled_ = ‐0.15, p = 0.02) and significant interactions between BAG and time since baseline visit (β_scaled_ = ‐0.02, p = 0.04) as well as amyloid positivity and time since baseline visit (β_scaled_ = ‐0.12, p < 0.001) in the Knight ADRC. The baseline difference in PACC for amyloid positive participants equates to the same effect as roughly +15 year in BAG.

**Conclusion:**

In a cohort with extensive longitudinal follow‐up, we observed a decline in cognitive performance over time associated with both BAG and amyloid positivity. Amyloid positivity may have a stronger effect on cognitive performance because it represents a disease trajectory, while BAG may more closely approximate typical aging.